# PHaLIR: prevent hernia after loop ileostomy reversal—a study protocol for a randomized controlled multicenter study

**DOI:** 10.1186/s13063-023-07430-w

**Published:** 2023-09-08

**Authors:** Karolina Eklöv, Sven Bringman, Jenny Löfgren, Jonas Nygren, Åsa H. Everhov

**Affiliations:** 1 Department of Clinical Science and Education, Södersjukhuset, Karolinska Institute, Stockholm, Sweden; 2 Department of Surgery, Södertälje Hospital, Södertälje, Sweden; 3 Department of Molecular Medicine and Surgery, Karolinska Institute, Stockholm, Sweden; 4 Department of Surgery, Ersta Hospital, Department of Clinical Sciences, Danderyd Hospital, Karolinska Institutet, Stockholm, Sweden; 5 Clinical Epidemiology Unit, Department of Medicine Solna, Karolinska Institute, Stockholm, Sweden

**Keywords:** Loop ileostomy reversal, Hernia, Rectal cancer, Prevent hernia

## Abstract

**Background:**

Rectal cancer is a common cancer worldwide. Surgery for rectal cancer with low anterior resection often includes the formation of a temporary protective loop ileostomy. The temporary ostomy is later reversed in a separate operation.

One complication following stoma closure is the development of a hernia at the former stoma site, and this has been reported in 7–15% of patients. The best method to avoid hernia after stoma closure is unclear. The most common closure is by suturing only, but different forms of mesh have been tried. Biological mesh has in a randomized trial halved hernia incidence after stoma reversal. Biosynthetic mesh and retromuscular mesh are currently being evaluated in ongoing studies.

**Methods:**

The present multicenter, double-blinded, randomized, controlled study will compare standard suture closure of the abdominal wall in loop ileostomy reversal with retromuscular synthetic mesh at the stoma site. The study has been approved by the Regional Ethical Review board in Stockholm.

Patients aged 18–90 years, operated on with low anterior resection and a protective loop ileostomy for rectal cancer and planned for ileostomy reversal, will be considered for inclusion in the study. Randomization will be 1:1 on the operation day with concealed envelopes. The estimated sample size is intended to evaluate the superiority of the experimental arm and to detect a reduction of hernia occurrence from 12 to 3%. The operation method is blinded to the patients and in the chart and for the observer at the 30-day follow-up. The main outcome is hernia occurrence at the stoma site within 3 years postoperatively, diagnosed through CT with strain. Secondary outcomes are operation time, length of hospital stay, pain, and 30-day complications.

**Discussion:**

This double-blinded randomized controlled superiority study will compare retromuscular synthetic mesh during the closure of loop ileostomy to standard care. If this study can show a lower frequency of hernia with the use of prophylactic mesh, it may lead to new surgical guidelines during stoma closure.

**Trial registration:**

ClinicalTrials.gov NCT03720262. Registered on October 25, 2018.

**Supplementary Information:**

The online version contains supplementary material available at 10.1186/s13063-023-07430-w.

## Ethics and dissemination

The study has been approved by the Regional Ethical Review board in Stockholm (protocol no: 2017/1693-31/2), with amendment nos.: 2021-00818, 2019-01860 and 2022-05253-02. The results will be disseminated through conventional scientific channels.

## Strengths and limitations of this study

- This is to our knowledge the only randomized controlled study to investigate synthetic mesh to prevent hernia after loop ileostomy closure.
- The primary outcome of hernia will be measured objectively and is clinically relevant and important to patient and physician alike.
- The trial is double-blind.
- The planned sample size is adequate to evaluate the superiority of the intervention arm.

## Introduction

### Background and rationale

Rectal cancer is a common form of cancer worldwide, with an age-standardized incidence rate of 14.4–16.6 per 100,000 for men in Europe, Eastern Asia, and Australia [1]. A temporary protective loop ileostomy is widely used when operating rectal cancer with low anterior resection [2–5]. The purpose of this practice is to reduce the impact of possible anastomotic leakage, but the method is constantly under debate because of its complications [6]. Ostomies can reduce the quality of life by causing leakage, parastomal hernia, and prolapse [7–11].

A temporary ostomy is reversed in a separate operation. One complication in connection with stoma closure is the development of a hernia at the former stoma site. A hernia is a weakening of the layers of the abdominal wall [12], which may cause pain and discomfort, but also more serious complications such as obstructed or strangulated bowel. The incidence of hernia after ostomy closure varies between 7 and 35% [13–20], whilst in studies including only ileostomy reversal, the hernia incidence was 7–15% [16, 17, 19–21].

The best method to avoid hernia after stoma closure is unknown. Most commonly, surgeons close the aponeurosis in one layer with a monofilament suture [20]. The use of prophylactic mesh in the abdominal wall has been tried in smaller non-randomized studies [22–24]. A meta-analysis from 2021 including both ileo- and colostomies showed a reduction in hernia incidence from 18.1 to 7.8% with no increase in complications using different types of reinforcing mesh [25]. In a retrospective study from 2018, the use of synthetic mesh reduced the incidence of hernia from 17.2 to 1% [24].

In a retrospective comparative study reviewing only the closure of ileostomies, the hernia incidence was reduced from 36.1 to 6.4% with a synthetic onlay mesh [26]. A multicenter study, randomizing loop ileostomy reversal between biological mesh and standard closure, showed significantly lower proportions of stoma site hernia, 12% vs 20%, in the mesh group, without increasing the complication rate [27].

The use of synthetic mesh is a well-established method in hernia repair, but there are no guidelines for abdominal wall closure in stoma reversal, despite the large defect in the aponeurosis. The ongoing PRINCESS study evaluates biosynthetic mesh for loop ileostomy reversal [28]. No completed randomized studies have compared a synthetic mesh with standard closure, but the ongoing study PELION are planning to evaluate synthetic mesh for loop ileostomy closure [29]. If the present study can detect a decreased frequency of hernia occurrence when using prophylactic mesh, it may lead to new recommendations for this patient group.

## Objectives

We hypothesize that a synthetic mesh placed retromuscularly in the abdominal wall reduces the frequency of hernia at the stoma site without increasing the risks of infection or other complications.

- The primary objective is to study whether a retromuscular synthetic mesh at the stoma site gives fewer hernias at the stoma site compared to a standard closure.
- The secondary objective is to compare operation time, length of hospital stay, pain, and 30-day complications, between the two groups.

## Trial design

Prevention of hernia after loop ileostomy reversal (PHaLIR) is a non-commercial clinical trial with the purpose of finding an operating method that reduces the incidence of hernias without increasing complications in patients undergoing loop ileostomy closure.

PHaLIR is a prospective, parallel-group, double-blinded randomized study in which patients planned for stoma reversal after rectal cancer surgery will be randomly allocated to an intervention group (retromuscular mesh repair (Ultrapro Advanced™, Ethicon, Hamburg, Germany)) or the control group (sutured abdominal wall closure). The allocation ratio is 1:1. The groups are parallel and non-crossover and the framework in the primary objective is superiority.

## Methods: participants, interventions, and outcomes

### Study setting

This multicenter study will be conducted in Swedish hospitals where loop ileostomy reversal after low anterior resection of rectal cancer is performed.

The hospitals participating in the study are the following University hospitals and regional hospitals located in the Stockholm and the Mid-Sweden region.

South General Hospital

Södertälje Hospital

Ersta Hospital

S:t Göran Hospital

Karolinska University hospital

Västerås Central Hospital

Gävle Hospital

Torsby Hospital.

### Eligibility criteria

All patients from the included centers who fulfill the inclusion criteria are to be evaluated for participation in the study. Patients not included in the study will be registered in the screening log with reason for non-participation.

#### Inclusion criteria

- Patients undergoing low anterior resection for rectal cancer with a diverting loop ileostomy and planned for stoma reversal with suture of the aponeurosis according to standard routines.
- Age 18–90 years

#### Exclusion criteria

- Language barrier or cognitive disability
- Recurrent inoperable cancer (patients with operable metastasis are not excluded)
- Large parastomal hernia not suitable for standard closure

#### Trial center requirement

Eligibility criteria for surgeons are that at least one should be a specialist with experience of colorectal surgery.

Centers can participate provided that the center has one local investigator in charge and that loop ileostomy reversals after low anterior resection for rectal cancer are performed in the center. Where stoma reversal and rectal cancer surgery are performed in different hospitals, the center where stoma reversal is performed will be responsible for the randomization, operation, and 30-day visit, whereafter the primary referral center will be responsible for a 1 and a 3-year follow-up.

### Who will take informed consent?

The patients will be identified and asked about participation when they come for postoperative follow-up after rectal cancer surgery and will be planned for ileostomy reversal. Potential study participants will be given oral and written information by the responsible surgeon. Signed informed consent is required from all study participants.

### Additional consent provisions for collection and use of participant data and biological specimens

The study does not collect biological specimens and does not have any associated ancillary studies.

### Interventions

#### Explanation for the choice of comparators

Reversal of loop ileostomy is a common surgical procedure in colorectal departments. Standard treatment entails closure of the abdominal wall with PDS 2/0 monofilament in one layer.

#### Surgical methods

##### Preoperative measures

One dose of preoperative oral antibiotic is to be given in the morning of the operation day. The antibiotics used will be sulfametoxazol/trimetoprim 800 mg/160 mg 1 tablet, and Metronidazole 400 mg, 3 tablets. Antithrombotic prophylaxis should be given according to local routines. Enhanced recovery programs may be used wholly or partially, but the program chosen by a clinic should not change during the study period.

##### Operation description

On the day of surgery, the patient will be randomized to standard abdominal wall closure according to the surgeon’s preference; this in most cases will be closure with 2/0 absorbable monofilament in one layer, or placement of retromuscular mesh, Ultrapro-Advanced™, size 5 × 5 cm. The operation method in the intervention arm will be as follows:

1. A circular incision will be made around the stoma.
2. The stoma will be detached from the abdominal wall. A hand-sewn anastomosis using 4/0 absorbable monofilament in one layer, with seromuscular suture, will be performed.
3. The bowel will be reinserted in the abdominal cavity.
4. The posterior rectus aponeurosis (or the peritoneum depending on the placement of the stoma) will be mobilized from the surrounding tissue and sutured with a 2/0 slowly absorbable monofilament running suture with a start and a stop knot.
5. The 5 × 5 cm Utrapro Advanced mesh will be placed in the retromuscular space. It should fill the width of the sheath of the rectus muscle.
6. A widening of the incision crosswise will be permitted if there are technical difficulties.
7. The anterior rectus sheath will be closed with a 2/0 non-absorbable monofilament running suture with a start and a stop knot.
8. The skin will be closed with an intracutaneous purse-string suture using 3/0 or 2/0 non-absorbable monofilament. If the incision is extended, the extension will be closed with a 4/0 absorbable monofilament running intracutaneous suture prior to the purse-string suture.
9. Finally, 20 ml Marcain/adrenalin 5 mg/ml 20 will be injected.

#### Adherence assessment

No specific adherence assessment is planned.

#### Criteria for discontinuing or modifying allocated interventions

The following situations resulting in discontinuation may arise:

1. A patient may have been inadequately enrolled, not meeting the eligibility criteria. This patient should be excluded from data analysis and only reported in the flow chart.
2. If a patient dies or is lost to follow-up, the data can be used until the date of the last follow-up.
3. If a patient withdraws from the study, the study data collected until this point will be used.

#### Strategies to improve adherence to interventions

Patient adherence is expected to be good because the study follow-up will be done in conjunction with the routine cancer follow-up. The clinicians’ adherence will be continuously followed up with information and reminders.

#### Relevant concomitant care permitted or prohibited during the trial

All relevant concomitant care is permitted during the trial.

#### Provision for post-trial care

There will be no specific provisions for post-trial care. The patients will be treated according to established routines.

### Outcomes

#### Primary outcome

The primary outcome is a hernia at the former stoma site at any time point during the 3-year study follow-up. Hernia will be diagnosed through CT abdomen with and without strain, performed 1 and 3 years after cancer surgery. A patients’ questionnaire will be run at the same time.

#### Secondary outcome

The secondary outcomes are postoperative complications, length of stay, and operation time at the 30-day visit, assessed by clinical investigation, protocol, and questionnaire.

### Participant timeline

#### Entry procedures

Patients operated on for rectal cancer with low anterior resection with loop ileostomy will be screened for enrolment after rectal cancer surgery is performed and the patient is planned for loop ileostomy reversal.

#### Enrolment process

**Fig. 1:**
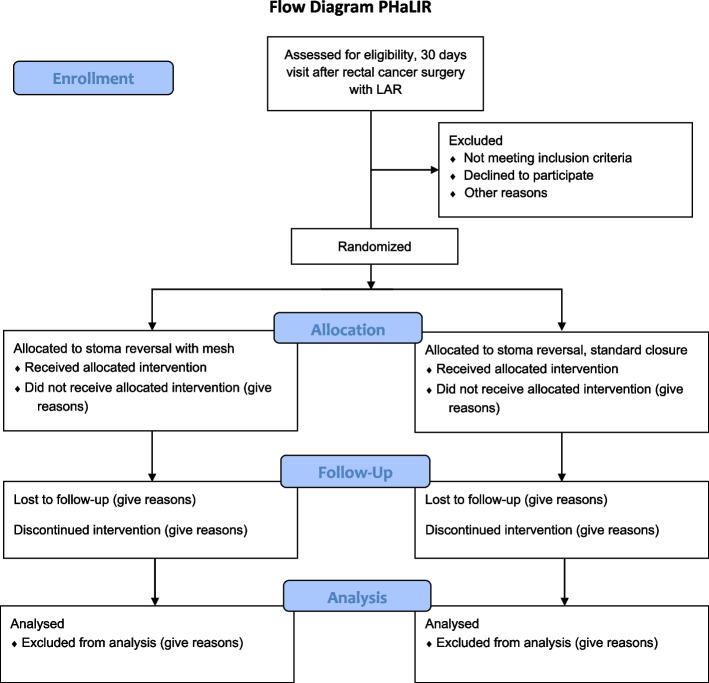
Flow chart of the study

A suitable time for enrolment is the 30-day follow-up after rectal cancer surgery. It is not mandatory to document the screening process, and the degree of screening may vary between participating centers (Fig. 1). Patients will be asked to participate by the surgeon responsible for the follow-up visit, will be given information, will sign a written consent, and will be given an enrolment number.

#### CRF (Case Report Form)

- Operation day: protocol will be filled out in connection with the operation and the operating notes blinded according to instruction.
- 30-day follow-up: patient questionnaire filled out by patient and protocol will be filled out by another doctor than the operating surgeon.
- 1-year and 3-year follow-up (in connection with cancer control): patient questionnaire and results from the CT scan.

### Sample size

Retrospective studies have shown a cumulative incidence of hernia between 7.4 and 23% after loop ileostomy reversal [16, 17, 19–21]. The reduction of hernia is described to be reduced up to 5.5 times [26] with a synthetic mesh and twice with a biological mesh [30]. We estimate a baseline cumulative hernia incidence of 12% and a triple reduction in hernia frequency using synthetic mesh. Some 208 patients (104 in each arm) are required in order to detect a reduction of hernia occurrence from 12 to 3% when using a double-sided *t-*test, a p-value of 0.05, and 80% power. We are planning to add 10% to compensate for loss to follow- up.

### Recruitment

Patients will be screened for participation in the study at the 30-day check-up after rectal cancer surgery.

## Assignment of interventions: allocation

### Allocation

The participants will be randomly assigned to either standard care or an experimental arm with a 1:1 allocation.

### Sequence generation

We will use computer-generated random sequence in batches of ten. We will not use stratification. The sequence allocation is done by KE and the research nurse.

### Concealment mechanism

The randomization will be done centrally in South General Hospital, and the notes with allocation group will be placed in opaque sealed envelopes and sent to the participating centers in batches of 10.

### Randomization

The randomization envelopes will be stored with the operation protocol and the instructions for the operation in a place convenient for each center. The envelope will be opened by the surgeon performing the operation on the operation day, before starting the operation.

### Implementation

The operation will be performed according to the randomization by the surgical specialists with colorectal profile at each hospital. The operation protocol will be filled in after surgery. The randomization instruction will be returned to the envelope with the operation protocol and stored in a closed locker. The operation notes in the patient chart will be blinded. The full version of the operation notes, including the abdominal wall closure, will be written on paper and stored until the study is finished and will then be added to the patient chart. The randomization number will be noted in the patient’s file.

## Assignment of interventions: blinding

### Who will be blinded?

1. The operation note will be blinded for the patient including the patient’s chart. The blinded part is the closure of the abdominal wall. After the bowel is returned to the abdominal cavity, the operation report will run as follows:
  “The patient has been randomized and the closure of the abdominal wall is according to the arm to which he/she has been assigned in the PHaLIR protocol.”
  The surgeon will document a short amendment containing the method of abdominal wall closure, i.e., either with or without mesh, in a separate Word document kept with the operation protocol and the randomization envelope after the operation.
2. At the 30 day-visit, the patient is to be seen by another surgeon than the operating surgeon. This is the blinded observer.

### Procedure for unblinding if needed

To break the code in an emergency, the local investigator is to be contacted. The list of personal identity and enrolment numbers will be stored in a closed locker in the research unit.

## Data collection and management

The following data will be collected and evaluated:

**Table:** 

• Age	operation protocol, surgeon
• Gender	operation protocol, surgeon
• Length	operation protocol, surgeon or anesthetist nurse
• Weight	operation protocol, surgeon or anesthetist nurse
• ASA class	operation protocol, surgeon or anesthetist nurse
• Smoking	operation protocol, surgeon
• Immunosuppression	operation protocol, surgeon
• Diabetes	operation protocol, surgeon
• Collagenous disease	operation protocol, surgeon
• Parastomal hernia preop	operation protocol, surgeon
• Operating time	operation protocol, surgeon
• Time for abdominal wall closure	operation protocol, surgeon
• Bleeding	operation protocol, surgeon or anesthetist nurse
• Bowel injury	operation protocol, surgeon
• Complication	30-days follow up, doctor
• SSI	30-days follow-up, doctor
• Postop hernia	30-day follow-up, doctor, 1 year, 3 years, CT
• Pain and inconvenience	patient, nurse, doctor on follow-up, questionnaires

### Plans for assessment and collection of outcomes

Data will be collected at four points.

1. The operation day of stoma reversal. The protocol is to be filled in by the operating surgeon after the operation.
2. Thirty-day visit at clinic. The doctor should be other than the operating surgeon. The doctor will fill in a 30-day protocol and the patients a questionnaire.
3. The 1-year and 3-year visits will be done at the same time as the standard visits for cancer follow-up. However, the surgeon responsible is to order the required CT abdomen *with strain* at 1 and 3 years. Patients are to fill in the questionnaires.

### Plans to promote participant retention and complete follow-up

Patients not responding on the questionnaire will be contacted by phone and the questionnaire sent again.

### Data management

Data will be stored in locked filing cabinets at the research unit at South General Hospital surgical clinic. The PI, research nurse, and local investigator at South General will have access to the data.

### Confidentiality

All data collection forms will be identified by a coded ID number only, to maintain participant confidentiality. All records that contain names or other personal identifiers, such as locator forms and informed consent forms, will be stored separately from study records identified by code number.

## Statistical methods

### Statistical methods for primary and secondary outcomes

Baseline characteristics between the intervention and non-intervention groups will be compared with paired tests. The incidence of hernia at the stoma site at 1 and 3 years will be compared between the two groups with paired tests and in a Kaplan-Meier model. Hernia in the abdominal wall is defined according to Muysoms [12].

If there are any imbalances between the two groups regarding factors known to influence the risk of hernia (e.g., sex and BMI), adjustment with regression analysis will be done. All data will be analyzed according to the intention-to-treat principle, as well as a per protocol analysis. Secondary outcomes will be compared between the groups by paired tests.

### Methods for any additional analyses

Not applicable. No additional analyses will be used.

### Interim analyses

No interim analysis is planned.

### Analysis methods to handle protocol non-adherence and any statistical methods to handle missing data

Data will not be imputed.

### Plans to give access to the full protocol, participant-level data, and statistical code

The full protocol will be disclosed on reasonable request. The dataset will not be publicly available due to patient privacy.

## Oversight and monitoring

### Composition of coordinating center and trial steering committee

The steering committee will meet twice yearly. The trial will be continuously overseen by the PI and the protocol committee.

### Composition of data monitoring committee, its role and reporting structure

No DMC is planned in this clinical study, using proven methods in adult patients.

### Plans for communicating important protocol amendments to relevant parties (e.g., trial participants, ethical committees)

During the process, more centers may be included in the study. For each of these, amendment is to be sent to the Ethics committee.

### Plans for collecting, assessing, reporting, and managing solicited and spontaneously reported adverse events

In case of adverse event, the patient will be cared for according to standard routines. If the adverse event is associated with the operation performed and information about the operation method is needed, the code will be broken according to the Procedure for unblinding. The study compares two established and widely used methods of surgery and will therefore not collect, assess, or report adverse events except register complications and treat them if needed according to clinical practice.

## Dissemination plans

The results will be published in peer-reviewed, scientific journals. Interested participants can get the reference to the relevant journal. The researchers responsible may be contacted by phone or e-mail for a summary of the findings.

## Discussion

This double-blinded randomized controlled study will compare the use of retromuscular synthetic mesh during the closure of loop ileostomy to standard care. The sample size is adequate to evaluate the superiority of the experimental arm. If this study can show a lower frequency of hernia with the use of prophylactic mesh, it may lead to new surgical guidelines during stoma closure.

## Trial status

Protocol version 1. Date of recruitment start 1 March 2018. Date of recruitment completed preliminarily Dec. 2024.

### Supplementary Information

**Additional file 1.** Patient consent.

## Data Availability

The PI and the protocol committee will have access to the final trial dataset.

## Abbreviations

GCP course: Course in Good Clinical Practice
CT: Computed tomography
DMC: Data monitoring committee
PI: Principal investigator
MDC: Multidisciplinary Conference
CRF: Case Report Form
