# Supplementary material for: PHaLIR: prevent hernia after loop ileostomy reversal—a study protocol for a randomized controlled multicenter study
Source: Trials. 2023 Sep 8;24:575. doi: 10.1186/s13063-023-07430-w (PMC10486037; doi:10.1186/s13063-023-07430-w)
Supplement: Supplementary file 1 — Additional file 1. Patient consent. [file 13063_2023_7430_MOESM1_ESM.docx]

**Kod_____________________ Datum_______________________**

**Patientinformation PHaLIR, 2 ex**

Till dig som tidigare genomgått uppläggning av stomi

**Du tillfrågas härmed om att delta i en studie:**

Retromuskulärt nät för att förebygga bråck efter stominedläggning, PHaLIR

**Bakgrund**

I ärret efter tidigare operationer samt även efter nedläggning av stomier förekommer det att man senare utvecklar ett bråck på denna plats med symtom av obehag, buktning samt smärta. Inläggning av nät bakom bukmuskeln vid bråckoperation är en väletablerad operationsmetod med goda resultat som används på Ersta sjukhus, Södertälje sjukhus, Södersjukhuset liksom på många andra sjukhus. Det har föreslagits att man skulle kunna lägga in ett nät i samband med stominedläggningen för att på det sättet undvika bråck på detta ställe. Dock finns det idag inte tillräckligt många studier som på ett vetenskapligt tillfredsställande vis jämför att lägga ner stomi utan nätinläggning respektive med nätinläggning.

**Syfte med studien**

Syftet med vår studie är att jämföra två olika operationsmetoder vid nedläggning av loop-ileostomi: sedvanlig nedläggning med slutning av tarmen och förslutning av bukväggen med tråd, eller slutning av tarmen och förslutning av bukväggen med nätinläggning. Du kommer att bli lottad till en av metoderna och det kommer vara okänt för dig vilken metod det blir. Resultatet av de olika operationsmetoderna jämförs med avseende på bråckförekomst, operationstid, vårdtid, infektioner, övriga komplikationer samt patientens upplevelse av operationsresultatet. På så sätt vill vi skapa ett vetenskapligt underlag för att i framtiden bättre kunna rekommendera optimal operationsmetod samt i bästa fall minska lidande pga framtida bråck.

**Förfrågan om deltagande**

Du har fått det här informationsbladet eftersom du planeras för nedläggning av loop-ileostomi och därför skulle kunna delta i vår studie. Medverkan i studien är helt frivillig. Skulle du tacka ja till att medverka kan du ändå när som helst, och utan att behöva ange varför, avsluta ditt deltagande utan att detta på något vis påverkar din vård i övrigt.

**Hur går studien till?**

Samtliga patienter som opererats för rektalcancer och är aktuella för nedläggning av stomin vid de aktuella sjukhusen får denna information. Bifogat finns en blankett för registrering av samtycke till att delta. Om Du, efter att ha läst denna patientinformation, accepterar att delta i studien ber vi dig att underteckna samtyckesblanketten.

**Vilken operationsmetod är bäst?**

I nuläget finns ingen säker kunskap om vilken operationsmetod som är att föredra. Genom att ta reda på vilken operationsmetod som leder till nöjdast patienter och minst risk för nya operationer räknar vi med att kunna utveckla bättre behandlingsmetoder i framtiden.

**Risker och obehag**

Deltagande i studien innebär inga särskilda risker utöver normala operationsrisker. Operation med nätinläggning beräknas ta max ½ timme längre tid än den andra operationen.

**Finns det några fördelar?**

Om du väljer att delta i studien bidrar du till ökad kunskap kring dessa två olika operationsmetoder. Ditt deltagande innebär inga direkta fördelar bortsett från att du, i samband med återbesök, får en något mer omfattande uppföljning av det genomförda ingreppet.

**Hantering av personuppgifter och sekretess:**

Vid analyser och resultatbearbetning kommer namn och personnummer på studiedeltagarna att ersättas med en kod så att någon enskild individ inte kan urskiljas. Endast den som är ansvarig för studien har tillgång till ”kodnyckeln”. Dina resultat kommer alltså att behandlas så att inte obehöriga kan ta del av dem. Det avkodade materialet kan eventuellt komma att användas av andra forskare inom EU. När resultaten från studien redovisas i vetenskapliga tidskrifter kommer enskilda individer inte att kunna identifieras.

Hanteringen av dina uppgifter regleras av GDPR **(GDPR) (EU 2016/679)** Ansvarig för behandling av dina personuppgifter är Södersjukhuset. Du kan vända dig till Södersjukhusets personuppgiftsombud om du önskar utdrag över de personuppgifter som finns registrerade på dig. Se nedan.

**Hur får jag information om studiens resultat?**

Inga individuella resultat kommer att finnas tillgängliga. Studiens resultat kommer att publiceras i vetenskaplig tidskrift efter ca 3 år. Intresserade forskningspersoner kan erhålla referens till aktuell tidskrift.

**Försäkring**

Deltagande patienter omfattas, i samma utsträckning som andra patienter, av patientskadeförsäkringen.

**Ekonomisk ersättning**

Ingen ekonomisk ersättning utgår.

## Frivillighet

Deltagande i forskningen är helt frivilligt. Du kan när som helst och utan förklaring återta ditt samtycke och avbryta studiedeltagandet utan att det påverkar övrigt omhändertagande

Om du har frågor eller vill ha mer information om studien är du välkommen att kontakta någon av nedanstående

**Projektansvariga**

**Karolina Eklöv** **Jonas Nygren** **Sven Bringman**

Biträdande överläkare Överläkare/docent Överläkare/Docent

Kirurgkliniken Kirurgkliniken Kirurgkliniken

Södersjukhuset Ersta sjukhus Södertälje sjukhus

116 28 Stockholm 116 28 Stockholm 116 28 Södertälje

Tfn 08-6161000 (vxl) Tfn 08- 714 65 00 (vxl) Tfn 08-55024000

Mobil 073-7008675

Victoria Wocalewski Forskningsssk Ssk Sofhie Sköld Cecilia Lindberg

08-6162695 08-7146537 08-55024100

Samtycke till

- ***Att delta i studien***

Retromuskulärt nät för att förebygga bråck efter stominedläggning

- ***Att personuppgifter registreras i enlighet med*** ***GDPR* (EU 2016/679)**

Jag samtycker till att de personuppgifter som angivits i studieinformationen samlas in och behandlas i enlighet med GDPR. Ansvarig för personuppgifterna är Södersjukhuset och dess Dataskyddsombud. Kontaktperson är projektansvarig läkare. Efter skriftlig begäran kan ett registerutdrag med de personuppgifter som registrerats lämnas ut kostnadsfritt och du har rätt att få information om vilka personer som haft åtkomst till dina uppgifter. Framkommer det att det står något felaktigt ska den felaktiga uppgiften rättas. Det är helt frivilligt att lämna uppgifter och du kan när som helst avbryta deltagandet. Redan insamlade uppgifter får dock behållas. Då forskning betraktas som allmänt intresse så är det den rättsliga grunden för hantering av personuppgifter. Du har rätt till skadestånd om personuppgifterna hanteras i strid med GDPR. Personuppgifterna kommer att sparas i 10 år efter att studien avslutats.

*För mer information se*[*www.datainspektionen.se*](http://www.datainspektionen.se/)

## Att studiepersonalen har tillgång till min journal

Jag samtycker till att forskningsenhetens personal under studietiden har tillgång till min journal på de aktuella sjukhusen. Mina uppgifter behandlas i enlighet med offentlighets- och sekretesslagen (2009:400) och patientsäkerhetslagen (2010:659). *För mer information se* [*www.socialstyrelsen.se*](http://www.socialstyrelsen.se)

*Projektansvarig läkares namn, telefonnummer och adress finns i studieinformationen.*

.........................……………. ..............................................................……………………...

Datum Forskningspersonens underskrift

........................................................................................

Forskningspersonens namnförtydligande

.......................................... ____________________________________________________

Datum Läkarens underskrift

.............................................................................................

Läkarens namnförtydligande
